# Hyperbaric oxygen therapy for a refractory skin ulcer after radical mastectomy and radiation therapy: a case report

**DOI:** 10.1186/s13256-016-1168-0

**Published:** 2017-01-04

**Authors:** Mitsuhiro Enomoto, Kazuyoshi Yagishita, Kae Okuma, Takuya Oyaizu, Yasushi Kojima, Atsushi Okubo, Takuma Maeda, Satoko Miyamoto, Atsushi Okawa

**Affiliations:** 1 Hyperbaric Medical Center, University Hospital of Medicine, Tokyo Medical and Dental University, 1-5-45 Yushima Bunkyo, Tokyo, 113-8519 Japan; 2 Center for Sports Medicine and Sports Dentistry, Tokyo Medical and Dental University, 1-5-45 Yushima Bunkyo, Tokyo, 113-8519 Japan; 3 Department of Radiology, University of Tokyo Hospital, 7-3-1, Hongo, Bunkyou-ku, 113-8655 Tokyo, Japan; 4 Department of Orthopedic and Spinal Surgery, Graduate School, Tokyo Medical and Dental University, 1-5-45 Yushima Bunkyo, Tokyo, 113-8519 Japan

**Keywords:** Mastectomy, Radiation injury, Refractory skin ulcer, Hyperbaric oxygen therapy

## Abstract

**Background:**

Radiation therapy is performed as an adjuvant therapy when indicated following surgical resection of malignant tumors. However, radiation exposure induces acute or chronic dermatitis, depending on the radiation dose, interval, tissue volume, or irradiated area of the body. Radiation-induced skin ulcers and osteomyelitis of the underlying bone are intractable late-stage complications of radiation therapy, and often require reconstructive surgery to cover exposed tissue. Hyperbaric oxygen therapy has been suggested as a treatment for delayed radiation injury with soft tissue and bony necrosis.

**Case presentation:**

A 74-year-old Japanese female underwent left radical mastectomy for breast cancer (T3N3M0, stage IIIB) in 1987. Radiation therapy was initiated 6 weeks after the surgery. She received telecobalt-60 in a total dose of 50 Gy with 25 fractions to the left supraclavicular, parasternal and left axillary regions, and electron treatment (9 MeV) in a total dose of 50 Gy in 25 fractions to the left chest wall. After irradiation, her skin became thinner and more fragile on the left chest wall, but no severe infections were observed. She noticed a small ulcer that repeatedly healed and recurred in 2000. She visited the hospital where she received radiation therapy and was treated for a skin ulcer on the left chest wall in December 2012. A fistula developed and then pus was discharged in January 2013. She was referred to the hyperbaric medical center in February 2013, and the fistula (1.5 × 3 cm) with pus discharge was observed. She was diagnosed with a late-onset radiation-induced skin ulcer that developed 25 years after radical mastectomy. HBO_2_ (2.5 atmospheres absolute with 100% oxygen for 60 minutes) was indicated for the refractory ulcer and osteomyelitis of the ribs. The patient was treated with HBO_2_ a total of 101 times over the course of 1 year and completely recovered.

**Conclusions:**

Hyperbaric oxygen therapy can be performed safely for even more than 100 sessions in patients with radiation-induced skin ulcers and osteomyelitis. Hyperbaric oxygen therapy can be considered as an alternative, conservative treatment when surgical resection for late-onset, radiation-induced skin ulcers is not indicated because of fragile skin in the irradiated areas.

## Background

Recently, the use of fluoroscopic procedures has increased as a result of advances in interventional radiology such as cardiac catheterization or intravascular surgery, which use such procedures. However, radiation exposure can cause dermopathies, including refractory skin ulcers [1]. Guidelines for radiation exposure were established by the Society of Interventional Radiology in 2009 [2]. Japanese guidelines were also established by the Japanese Society for Therapeutic Radiology and Oncology in 2012 (http://www.jastro.or.jp/guideline/child.php?eid=00007). Radiation therapy is widely recognized as a curative treatment for multiple types of cancers, including prostate, head and neck, lung, uterine, esophageal, and brain cancers, as well as lymphomas. In breast cancer, radiation therapy is used primarily as an adjuvant treatment following surgery [3]. However, high-dose radiation exposure of thin soft tissue often causes acute or delayed skin ulcers. In addition, osteomyelitis of the ribs becomes intractable when chest skin ulcers are present. In such cases, chest wall reconstruction might finally be needed in addition to aggressive skin or rib resection [4].

Hyperbaric oxygen therapy (HBO_2_) has been suggested for treatment of delayed radiation injury with soft tissue and bony necrosis [5]. We report a case of a patient with refractory skin ulcer of the left chest wall that developed after radical mastectomy and radiation therapy for breast cancer, which was successfully treated with HBO_2_.

## Case presentation

A 74-year-old Japanese woman underwent left radical mastectomy in 1987 for breast cancer with a size of 6 × 4 cm in the upper outer quadrant (T3N3M0, stage IIIB). Tamoxifen was administered in addition to tegafur and OK-432 as an adjuvant chemotherapy after the surgery. The tumor was negative for both estrogen receptor and progesterone receptor and was evaluated as N2b. Radiation therapy was initiated 6 weeks after the surgery. Initially, the patient received telecobalt-60 treatment, comprising 24 Gy in 12 fractions (days) to the supraclavicular and parasternal regions and 14 Gy in 7 fractions (days) to the left axilla, because of delayed wound healing after the surgery. Her wound was completely healed 15 days after the radiation treatment. The cobalt-60 treatment was resumed, comprising 26 Gy in 13 fractions to the supraclavicular and parasternal regions and 36 Gy in 18 fractions to the left axilla for 27 days, resulting in a total of 50 Gy in 25 fractions in each region. At the same time, electron treatment (9 MeV) to the left chest wall was started such that it did not overlap with the treatment of the supraclavicular, parasternal, and axillary regions. After the patient had received 44 Gy in 22 fractions at 32 days, skin redness and swelling occurred, and the treatment was stopped. After a 1-month rest period, 3 additional fractions were performed to provide a total of 50 Gy in 25 fractions. At that time, a keloidal change corresponding to the electron treatment area on the patient’s left chest wall was noted; her skin became thinner and more fragile, but no severe infections were observed.

**Fig. 1:**
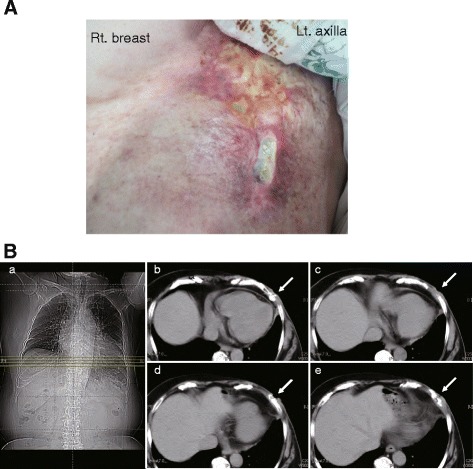
Gross appearance of the affected area and computed tomographic images of the left chest wall at the first visit. **a** A skin ulcer with redness and pus discharge was observed. **b** Computed tomographic images showing that the fistula did not connect into the thorax (*arrows*)

The patient noticed a small ulcer at the beginning of 2000, but the ulcer healed over time without treatment. She visited the hospital where she received radiation therapy in December 2012, and at that time she was treated with cadexomer iodine ointment for a skin ulcer on the left chest wall. A fistula eventually developed, and pus discharging from the fistula was observed in January 2013. She was referred to the hyperbaric medical center in February 2013. The fistula (1.5 × 3 cm) with pus discharge on her left chest wall was noted at her first visit (Fig. 1a). The skin around the lesion showed redness and swelling with a keloidal change. The pus discharge was synchronized to her respiratory rhythm, and a bacterial identification test result was positive for *Staphylococcus aureus*. A pulmonary function test revealed pulmonary restriction (percent predicted vital capacity [%VC] 60.6 %, percent predicted forced expiratory volume in 1 second [FEV_1%_] 80.2 %). Chest radiography showed cardiac enlargement. Chest computed tomography (CT) showed no cystic lesions, and the fistula located on the left chest wall did not invade the thorax (Fig. 1b). The patient was diagnosed with a late-onset, radiation-induced skin ulcer that developed 25 years after radical mastectomy. HBO_2_ was indicated for the refractory ulcer and osteomyelitis of the ribs. Her medical history included acute myocardial infarction and a stent implanted in 2003. She was instructed to communicate any problems including otalgia, ear fullness, hearing loss, tinnitus, vertigo, and dizziness to a physician at the hyperbaric medical center.

**Fig. 2:**
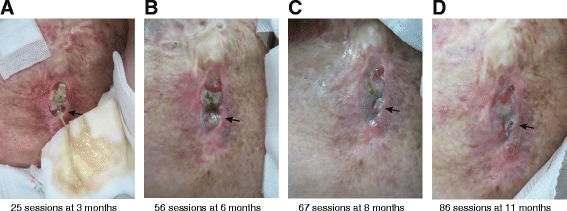
Representative gross appearance of the affected skin during hyperbaric oxygen therapy. **a** Massive pus discharge (*arrow*) was observed after 25 sessions. **b**–**d** Pus discharge and fistula size gradually decreased over time (*arrows*)

**Fig. 3:**
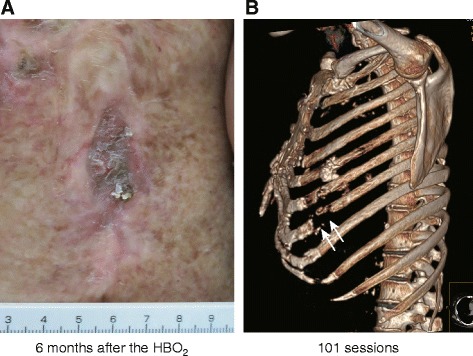
Gross appearance of the affected area and three-dimensional computed tomographic image of the left chest wall 6 months after starting hyperbaric oxygen therapy (HBO_2_). **a** The fistula completely disappeared. **b** Three-dimensional computed tomographic image showing a rib defect (*arrows*)

HBO_2_ therapy was started in March 2013 after written informed consent was obtained. A multiplace hyperbaric chamber (NHC-412-A; Nakamura Tekko-Sho K.K., Tokyo, Japan) was pressurized up to 2.5 atmospheres absolute (ATA) with 100 % oxygen delivered through a mask. The duration of each treatment session was 105 minutes, including 15 minutes of compression at the beginning and 20 minutes of decompression at the end of treatment. The patient tolerated the HBO_2_ protocol without any adverse effects, despite her pulmonary restriction and cardiac enlargement. After 25 sessions over the course of 3 months, granulation tissue was observed around the margin of the ulcer, but massive pus discharge was still noted (Fig. 2a). At this time, a physician suggested that the ulcer be washed with tap water once daily. Granulation tissue gradually increased and pus discharge diminished without use of antibiotics (Fig. 2b, c). After 86 sessions over the course of 11 months, epithelialization was observed and pus discharge disappeared (Fig. 2d). A pulmonary function test at this time still revealed pulmonary restriction (%VC 58.6 %, FEV_1%_ 85.2 %), with no remarkable change compared with previous findings. After 101 sessions over a period of 1 year, the ulcer was considered to be healed. The patient no longer had to cover her skin wound and was able to bathe after the treatment. At a follow-up visit 6 months after the completion of HBO_2_ therapy, no recurrence of skin ulceration was noted (Fig. 3a). Chest CT was performed to evaluate the further development of abnormal lesions. We found that osteomyelitis of the ribs had developed following the formation of the skin ulcer compared with the previous CT. A rib defect was observed on three-dimensional chest CT (Fig. 3b). The rib defect was managed conservatively because the patient’s symptoms were limited to left chest pain in her daily life.

## Discussion

Acute- and late-onset skin reactions are adverse effects of radiation therapy and are categorized using several scoring systems [6, 7]. Delayed changes usually develop within 4–6 months, and the irradiated skin could have an increased risk of infection, delayed healing, wound dehiscence, fistula formation, and wound necrosis [8]. Radiation-induced skin ulcers can become intractable as a result of improper wound healing, infection, or both. In our patient, thinning of the chest wall by resection of breast muscle and the irradiation protocol contributed to the formation of an intractable ulcer. According to the patient’s history of dermatitis during radiation therapy, the skin ulcer recurred 25 years after treatment. The patient’s skin was already red and fragile, and the ulcer had become intractable, but the infection was localized around the ulcer before her hospital visit. For these reasons, HBO_2_ was considered to be the best conservative treatment option. In the case of large skin ulcers with massive infection and bone necrosis, surgical treatment, including resection of the ulcer, sternum, and ribs with osteomyelitis followed by reconstruction of the chest wall using skin or muscle flaps, should be considered [9, 10]. Malignancy also should be considered in the differential diagnosis, and a biopsy should be performed to rule out a second malignancy in the radiated field [11]. In our patient, carcinogenesis was not suspected on the basis of the gross appearance of the skin and radiographic findings before starting HBO_2_. We examined the appearance of the skin at least every 2 weeks during the HBO_2_ sessions to detect nontypical wound healing.

HBO_2_ involves breathing pure oxygen at an ambient pressure higher than atmospheric pressure. The worldwide standard HBO_2_ protocol is to apply 2.0–2.8 ATA for 60–90 minutes with pressurized air or oxygen inside the chamber [12]. HBO_2_ is thought to have complex effects on immunity, oxygen transport, and hemodynamics, resulting in favorable therapeutic effects by reducing hypoxia and edema as well as enabling normal host responses to infection and ischemia [12, 13]. Indications for HBO_2_ according to the Undersea and Hyperbaric Medical Society include arterial insufficiency (enhancement of healing for select problematic wounds); refractory osteomyelitis; and delayed, radiation-induced soft tissue injury or bone necrosis [14]. There are several contraindications for HBO_2_. Absolute contraindications include doxorubicin treatment (because of cardiac toxicity), mafenide acetate treatment (because of central vasoconstriction), and untreated pneumothorax [15]. According to authors of a Cochrane review of chronic wounds, HBO_2_ improved healing in the short term but not with long-term follow-up [16]. In experimental studies, HBO_2_ treatment led to proliferation of bovine aortic endothelial cells and human skin fibroblasts in vitro as well as accelerated wound epithelialization and neovascularization in mouse ears [17, 18]. Clinically, a positive correlation has been reported between tissue oxygenation and various markers of wound healing. These results support the efficacy of HBO_2_ [19].

Our patient’s case met the indications for HBO_2_, and she had no recurrence at least 6 months after treatment. The average total number of HBO_2_ sessions for chronic wounds is in the range of 30–40 [20]. Our patient underwent 101 sessions over a period of 1 year. However, cases of radiation-induced injury require a greater number of sessions (30–60) than those involving chronic wounds [5]. In particular, radiation-induced ulcers of the chest wall with bone necrosis often require more frequent HBO_2_ sessions as well as surgical intervention [20]. In one case report, necrotic lesions induced by accidental radiation exposure improved after 140 sessions of HBO_2_, without any adverse effects [21]. Thus, delayed, radiation-induced injury of the chest wall can be treated successfully with more than 100 sessions of HBO_2_. However, long-term studies are needed to determine whether surgical intervention will eventually be required.

## Conclusions

We treated a patient with refractory skin ulcer on the left chest wall that developed 25 years after radical mastectomy and radiation therapy for breast cancer. The patient was successfully treated with HBO_2_ a total of 101 times over the course of 1 year without any adverse effects.

## Abbreviations

ATA: Atmospheres absolute
CT: Computed tomography
FEV_1%_: Percent predicted forced expiratory volume in 1 second
HBO_2_: Hyperbaric oxygen therapy
%VC: Percent predicted vital capacity
